# Second harmonic generation and broad-band photoluminescence in mesoporous Si/SiO_2_ nanoparticles

**DOI:** 10.1515/nanoph-2024-0218

**Published:** 2024-08-01

**Authors:** Viktoriia Mastalieva, Vladimir Neplokh, Arseniy Aybush, Ekaterina Stovpiaga, Daniil Eurov, Maksim Vinnichenko, Danila Karaulov, Demid Kirillenko, Alexey Mozharov, Vladislav Sharov, Denis Kolchanov, Andrey Machnev, Valery Golubev, Alexander Smirnov, Pavel Ginzburg, Sergey Makarov, Dmitry Kurdyukov, Ivan Mukhin

**Affiliations:** Alferov University, Khlopina 8/3, 194021, St. Petersburg, Russia; Ioffe Institute, Polytechnicheskaya Str., 26, 194021, St. Petersburg, Russia; Peter the Great St. Petersburg Polytechnic University, Polytechnicheskaya 29, 195251, St. Petersburg, Russia; N.N. Semenov Federal Research Center for Chemical Physics, Russian Academy of Sciences, Kosygin Street 4, 119991 Moscow, Russia; Tel Aviv University, Ramat Aviv, 69978 Tel Aviv, Israel; Qingdao Innovation and Development Center, Harbin Engineering University, Qingdao 266000, Shandong, China; ITMO University, 197101, St. Petersburg, Russia

**Keywords:** nonlinear nanophotonics, second harmonics, nanoparticles, silicon, broad-band photoluminescence

## Abstract

Efficient second harmonic generation and broad-band photoluminescence from deeply subwavelength and nontoxic nanoparticles is essential for nanophotonic applications. Here, we explore nonlinear optical response from mesoporous Si/SiO_2_, SiO_2_, and Si nanoparticles, considering various fabrication and treatment procedures. We show that thermal annealing (including femtosecond laser treatment) of mesoporous Si/SiO_2_ nanoparticles provides the transformation of Si phase from amorphous to crystalline, enhancing the second harmonic and nonlinear photoluminescent response. Notably, the SiO_2_ mesoporous frame of the considered Si/SiO_2_ nanoparticles plays a dual positive role for the nonlinear process: it stabilizes the Si material, and SiO_2_:OH^−^ material has a second-order nonlinearity itself and impacts to the observed second harmonic signal.

## 1 Introduction

Second-order optical nonlinearity is a well-known mechanism for modulating and generating coherent light in photonic devices and bio-imaging applications. Due to strong photon confinement and long photon lifetime, integrated microresonators have emerged as a promising platform for the investigation of nonlinear optical effects [1], [2], [3], [4]. Optical microresonators provide an implementation of efficient nonlinear effects at the micro- and nanoscale. However, existing silicon-based structures (including SiN_*x*_ and SiO_*x*_) still require the development of fabrication methods and nanoscale design since bulk silicon has a near-zero second-order response due to its centrosymmetric structure; thus, the second harmonics generation (SHG) efficiency is governed by the properties of Si surface. A variety of novel material platforms possessing high *χ* ^(2)^ nonlinearity have been developed over the past two decades [5], [6], [7], [8]. These studies carry a high value for fundamental science [8], [9], [10], [11] as well classical and quantum applications [12], [13], quantum communication and computation [14], [15], [16], [17], [18], [19], [20], [21], long-haul communication systems [18], infrared light visualization, etc. [19], [20], [21]. Moreover, optical micro- and nanoresonators help to overcome the requirement of strong phase matching for efficient SHG generation in bulk materials by replacing them by a modest condition of quasi-modal overlap [22].

AlGaAs is considered a versatile platform for efficient SHG generation [23], [24]. Among semiconductor materials, AlGaAs has one of the highest values of *χ* ^(2)^ nonlinearity and refractive index. However, it is not a trivial task to fabricate AlGaAs-based nanoparticles or nanocylinders on optically transparent wafers. Expensive epitaxial techniques are normally employed, which also require an additional layer having a higher content of Al with further oxidation to AlO_*x*_ to achieve optical separation (decoupling) of active (Al)GaAs layer (with lower Al content) and GaAs wafer.

At the same time, SiO_*x*_ and Si-based nanoparticles can be synthesized using cost-efficient colloidal approaches with a control of mesoporosity and ability to fill the pores with the required materials. In the early 1990s, porous Si emerged as a suitable platform for photonics [25]. Since the porous Si structure can provide a very high specific surface area above 100 m^2^/g [26], [27], surface-related SHG in mesoporous Si has been extensively studied in the last decades [28], [29], [30]. On the other hand, Si microresonators such as nanowires and nanoparticles are also marked by prominent second-order nonlinear properties [18], [19], [30], [31]. The electromagnetic field matching of pump irradiation and the generated second harmonic (SH) in nanoscale resonators [19], [32]–[35] leads to the elevated conversion efficiency compared to thin films and allows tuning of the nonlinear system properties, including the directivity of SHG emission [36], [37], [38], [39]. In other words, the effective volume of nanoresonators can be multiplied by a quality factor of optical mode. Thus, deeply subwavelength Si nanostructures with a nanocrystalline internal structure and high surface area properly passivated should further improve the efficiency of SHG and other nonlinear processes.

Concerning Si-based SHG structures, the mesoporous spherical particles [19] appear to be the ideal material system. According to our previous report [24], for these nanoparticles: (i) literally all Si material is a surface, no Si material is thick enough to be considered as bulk; (ii) synthesis method can be adjusted to any desired particle size in the range of few tens to several thousands of nanometers, while preserving the quality of mesoporous structure; (iii) diameter of pores is tunable in the range of 2–20 nm; thus, (iv) particles can be filled with beneficial material that further enhance SHG efficiency or related properties (refractive index, light absorption, control of surface electron states etc.); (v) synthesis method based on organic micelles provides a construction set for homogeneous arrays of Si particles with selected morphology, so they can be arranged in a 2D or 3D periodic grid (photonic crystal or synthetic opals [40], [41]) having linear optical properties differ from single nanoparticles or their chains and metasurfaces [42], [43], which can be useful for nonlinear response tailoring.

In this work, we for the first time propose mesoporous nanoparticles with a SiO_2_ frame filled with a Si phase, previously developed for drug delivery [26], for efficient SHG owing to high surface to volume ratio. By means of mapping in a IR-femtosecond (fs) laser scanning system, the SHG-to-pump characteristics were compared among mesoporous Si/SiO_2_, SiO_2_, and Si nanoparticles, considering various fabrication and treatment procedures. Thermal or laser-induced Si annealing leads to transformation of the Si phase from amorphous to nanocrystalline one, which improves the nonlinear performance of the studied nanoparticles. Moreover, the thermally treated Si/SiO_2_ nanoparticles also exhibit a broad-band photoluminescence under linear and fs-laser excitation, which can be employed for visualization in optics at the nanoscale.

## 2 Materials and methods

### 2.1 Synthesis of nanoparticles

Monodisperse spherical SiO_2_ nanoparticles (*reference SiO*_2_ *NPs*) with low porosity were produced through the hydrolysis of tetraethoxysilane (TEOS) in an alcohol-based solution containing ammonia and water [44], [45]. The primary substance concentration in the aqueous ammonia solution was 24 wt%. The initial alcohol concentration was 95.7 wt%. Both the alcohol and ammonia solutions were used as received. The synthesis process utilized deionized water with a specific resistance of 10 MΩ cm.

TEOS underwent fractional distillation with a boiling temperature of 166–168 °С [45], [46], [47]. This fraction was then treated with a 0.5 wt% aqueous ammonia solution for 20 min. The ratio of TEOS to the aqueous ammonia solution was 5:1 by mass (the procedure is detailed in Ref. [46]). The synthesis process had a duration of 4 h and resulted in a formation of 550 ± 20 nm monodisperse low-porosity (less than 15 %) nanoparticles having a high concentration of OH^−^ groups [26], [48]–[50]. The synthesized particles were centrifuged and annealed at 900 °C for 5 h.

**Figure 1:**
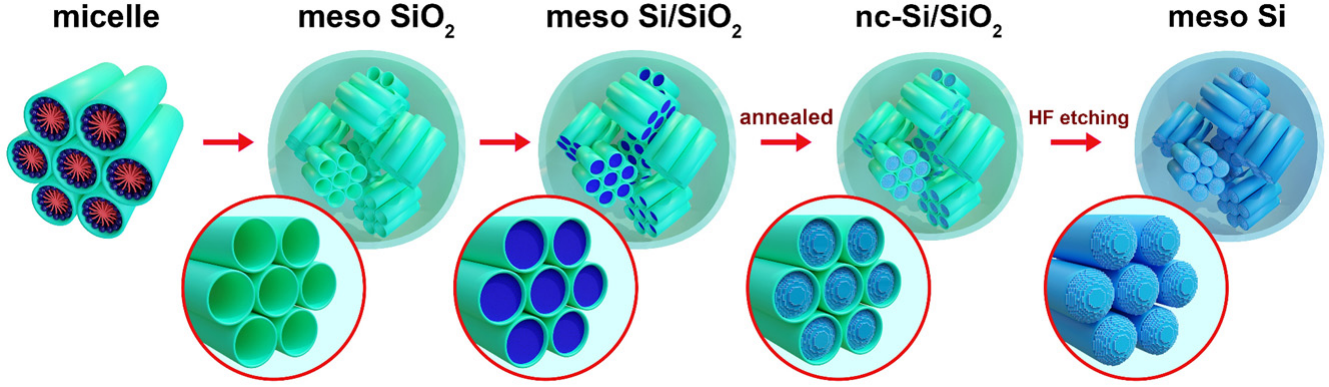
Fabrication scheme for mesoporous spherical SiO_2_ nanoparticles, hybrid Si/SiO_2_ NPs, and derived mesoporous Si nanoparticles.

Spherical mesoporous Si/SiO_2_ nanoparticles (*meso Si/SiO*_2_ *NPs*) were synthesized in several steps (see the workflow scheme in Figure 1). Monodisperse spherical mesoporous silica nanoparticles (*meso SiO*_2_ *NPs*) were synthesized according to our previously developed method [51] by hydrolysis of TEOS in a water–ethanol–ammonia medium containing cylindrical micelles of cetyltrimethylammonium bromide as a pore-forming agent. The silica particles obtained were centrifuged, dried in air at 70 °C for 24 h, and annealed at 550 °C for 5 h to remove micelles and form the pores. The specific surface area and pore volume of the particles synthesized were 810 m^2^/g and 0.55 cm^3^/g (about 50 % of particle volume), respectively, and the sectional diameter of pores was about 3 nm [51].

Silicon phase was synthesized within the pores of silica particles by the thermal decomposition of monosilane [26]. The synthesis was carried out in the reactor consisting of a fused silica tube equipped with an external resistive heater. A gas mixture of SiH_4_ (5 %) and Ar was passed through the tube at a flow rate of 1 sccm over a 1 g portion of the silica particles powder placed in a fused silica crucible. The thickness of the particle layer did not exceed 1 mm. The reactor pressure was 1,000 Torr. A constant temperature of 440 °C was maintained within the reactor, while the synthesis duration was 40 h. As a result of the thermal decomposition of silane, silicon filled the pores of meso SiO_2_ particles producing meso Si/SiO_2_ NPs. Note, the employed procedure provided the synthesis amorphous silicon phase filling the nanopores [26]. Additionally, to obtain meso Si/SiO_2_ NPs with nanocrystalline silicon (nc-Si) phase, the produced NPs were thermally annealed in an ampoule at a temperature of 850 °C (*annealed meso Si/SiO*_2_ *NPs*) [26].

The meso SiO_2_ NPs for the Si/SiO_2_ NP formation were found to have a diameter of 450 ± 30 nm [52]. The meso Si/SiO_2_ NP morphology was studied in [52], inherited from the micelle structure, the pores are clusters of 7 cylinders, hexagonally packed, with a diameter of ∼3 nm and a length of ∼10 nm (see Figure 1 schematics). The meso SiO_2_ NPs according to the porosimetry measurements had a porosity of 50 vol% [51]. This porosity allowed almost complete filling, so the synthesized meso Si/SiO_2_ NPs had about equal volume proportion of Si and SiO_2_ phases, and the remaining unfilled space inside the NPs was determined by comparing the sedimentation rates of unfilled and filled particles [50].

To obtain mesoporous Si nanoparticles (*meso Si NPs*), the SiO_2_ material was selectively etched out from the meso Si/SiO_2_ composite. For that purpose, the meso Si/SiO_2_ NPs were placed in a 0.015 M aqueous solution of hydrofluoric acid (HF). Etching was carried out for 10 h. The resulting Si particles were repeatedly washed with deionized water (10 MΩ cm) and sedimented by centrifugation.

### 2.2 Material characterization

Transmission electron microscopy (TEM) study was carried out to analyze the structural properties of the synthesized nanoparticles at the nanometer scale. The study was performed using JEM 2100F (Tokyo, Japan) high-resolution transmission electron microscopy (HRTEM) setup operating at 200 kV accelerating voltage.

Raman spectroscopy was employed to identify Si crystalline properties in meso Si/SiO_2_ NPs [53]. Raman spectra were acquired at ambient conditions using a Horiba Jobin Yvon T64000 (Japan) spectrometer, utilizing the second harmonic of an Nd:YAG laser (532 nm wavelength) as a light source. The spectrometer was equipped with a confocal microscope, allowing laser beam focusing into a spot of approximately 1.5 μm in diameter. The excitation density on the sample surface during Raman measurements did not exceed 1 kW/cm^2^. The spectrometer was calibrated using a reference Si (111) wafer.

Infrared (IR) transmission spectra were obtained at ambient conditions using a vacuum Bruker Vertex 80v (Germany) Fourier spectrometer operating in rapid-scan mode and equipped with a globar as a source of light, KBr beam splitter and pyroelectric photodetector with a CsI window. Spectral resolution was about 4 cm^−1^. Samples were mounted on a copper holder with an aperture of about 4 mm. To obtain the transmission spectra of the meso Si/SiO_2_ NPs, the intensity spectra of light passing through the Si/SiO_2_ NPs on the substrate were normalized to reference spectra of Si substrate.

### 2.3 Optical response and second harmonic measurements

For nonlinear optical measurements, the samples from an isopropanol colloid were applied onto precleaned tempered quartz holders to form several NP layers.

Nonlinear optical investigations were conducted using a confocal laser-scanning microscope (LSM) setup (LSM-980, Zeiss, Germany). The LSM external acousto-optic modulator (AOM) port was utilized to deliver femtosecond (fs) laser pulses (Discovery-NX, Coherent, USA) with the following parameters: (i) a repetition rate of 80 MHz, (ii) a duration of approximately 150 fs, and (iii) linear polarization. The tunable wavelength range for the pulses’ central wavelength of 800–1,020 nm (spectral full width at half maximum, FWHM < 10 nm) was limited by LSM optics. Si detector (S120C, PM100USB, Thorlabs, USA) was used to set the power value of laser irradiation applied to the sample, and it was calibrated for AOM levels of LSM. The maximum power of radiation can reach up to ∼25 mW (0.3 nJ per single pulse).

For the optical study, air microscopy objectives with numerical apertures of 0.3 and 0.8 (corresponding to magnifications of 10× and 20×, respectively) were employed. LSM measurements produced 16-bit images using a sensitive GaAsP photomultiplier tube detector, enabled by the galvo mirror system of LSM at an average speed of tens of mm/s (e.g., giving about 20 µs for a map pixel with a size of approximately 500 nm for the 20× objective). The nonlinear optical measurements were performed in a reflection mode, i.e., the focusing objective was also used to collect the optical response from the studied samples. The integrated SH signal was collected within a range of ±10 nm relative to the central wavelength of the harmonic for each pixel of the LSM image.

The spectral characteristics of the nonlinear response were obtained in the so-called “scanning Lambda LSM” mode with an optimal for the studied samples pump wavelength in the range of 900–980 nm, which allowed hyperspectral mapping in the visible range with a spectral resolution of 3 nm. For meso Si/SiO_2_ NPs, the spectral characteristics were obtained in the 425–700 nm range, demonstrating not only the SHG signal but also a broadband photoluminescence (PL) response.

## 3 Results and discussion

**Figure 2:**
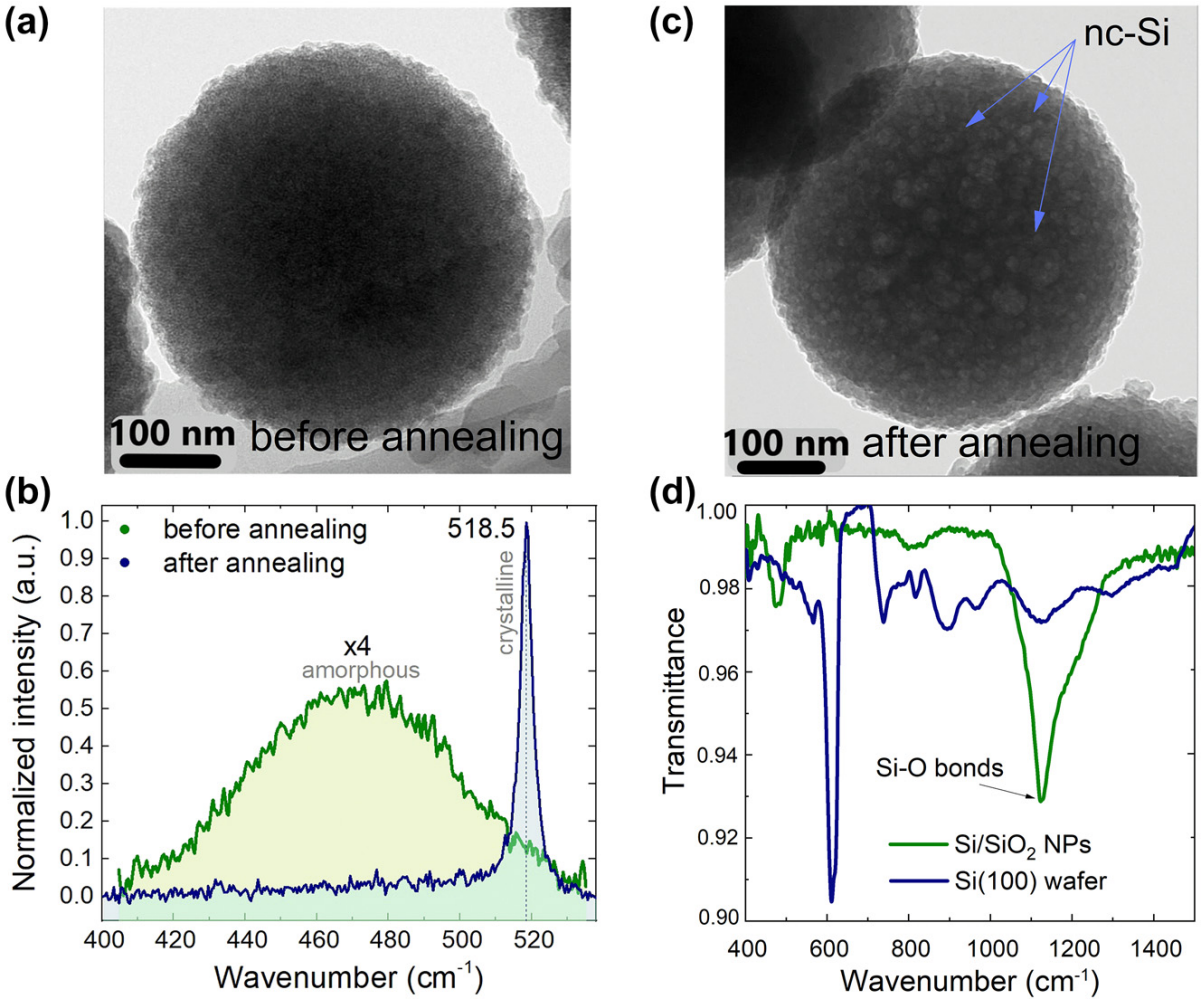
TEM images of meso Si/SiO_2_ NPs before (a) and after (b) thermal annealing at 850 °C. (c) Raman spectra of meso Si/SiO_2_ NPs before and after thermal annealing at 850 °C. For the convenience, the spectrum of as-synthesized particles is amplified by ×4 factor. (d) Transmittance spectrum of meso Si/SiO_2_ NPs sample after annealing (green line). Blue line corresponds to the spectrum of reference c-Si (100) wafer.

The performed scanning electron microscopy (SEM) imaging demonstrated no distinguishable difference between as-synthesized and annealed meso Si/SiO_2_ NPs (see Figure S1 in the Supporting Information), while TEM imaging for Si/SiO_2_ NPs before and after annealing revealed an enlarging of Si clusters that we attribute to improved crystallinity due to annealing (Figure 2a and b). Indeed, the obtained TEM images allow distinguishing two material phases; the nc-Si material is manifested by a bright contrast that is more pronounced after annealing.

Importantly, the SiO_2_ material in meso Si/SiO_2_ NPs had also a high OH^−^ group concentration (similar to reference SiO_2_ NPs). The Si material, however, appeared to be amorphous for the as-synthesized NPs [26], which was confirmed by Raman measurements (Figure 2c). Indeed, the representative Raman scattering spectrum for the particles before annealing did not demonstrate any clear line typical for the crystal phase [26], [54], while the observed weak Raman signal with a broad spectral band is typical for amorphous material [55]. After annealing of Si/SiO_2_ NPs, the acquired Raman spectra confirmed the presence of crystalline Si phase manifested by a prominent narrow line at 518.5 cm^−1^ corresponding to the transverse optical phonon mode of crystalline Si (Figure 2c) [56]. The 1.5 cm^−1^ shift relative to the bulk c-Si 520 cm^−1^ corresponds to the 5–10 nm size of nc-Si inclusions in accordance with our previously reported results [26] that also correlates to the data reported in the literature [57]. In Ref. [26], the volume fraction of the crystalline Si component relative to the amorphous component reaches 70 %.

IR transmission spectroscopy of meso Si/SiO_2_ NP samples provided essential information about the quality of the Si/SiO_2_ interface [58]. The absorption line at 1,080 cm^−1^ is characteristic for Si–O bonds (Figure 2d), and its presence confirms a high-quality interface in meso Si/SiO_2_ NPs strongly affecting SHG properties [53], [59], [60], [61]. Also peaks at 810 and 456 cm^−1^ could assign the symmetric stretching and rocking modes of the Si–O–Si vibrations in SiO_2_ phase [61]. For a comparison, Figure 2d shows the corresponding IR transmittance spectrum for a reference c-Si (100) substrate, confirming the absence of Si–O bonds.

According to the described synthesis procedure, the meso Si NPs were obtained from the meso Si/SiO_2_ NPs by SiO_2_ etching; thus, they keep the same diameter (450 ± 30 nm in our case). As shown in Ref. [26], after SiO_2_ removal, the Si material kept an amorphous structure and configuration of initial pore clusters. After annealing in the inert atmosphere of the sealed ampoules, the Si material transformed into nanocrystalline phase; however, the meso Si NP colloid oxidizes in the atmosphere in a matter of days. The dried meso Si NPs (e.g., transferred to quartz holders for the nonlinear measurements) acquire natural oxide surface layer in half an hour, that in case of mesoporous structure take a significant portion of the material volume (i.e., 0.5 nm thick shell for the 3.1 nm Si veins counts for about 54 % of their volume). Thus, these samples were characterized during 24 h after preparation, transported as a colloid, and placed to quartz holders just before SHG measurements. It should be noted that meso Si NPs turn to pure SiO_2_ in several weeks while being stored in an isopropanol colloid.

To summarize the material characterization results, we expected the strongest SHG signal from the annealed meso Si NP samples due to their structure being a practically pure nc-Si surface that is known to demonstrate the highest SHG efficiency among silicon and silica materials (without doping) [62]. A comparable SHG signal was expected for the annealed meso Si/SiO_2_ NP samples, because they also contain mesoporous nc-Si phase, but the SHG efficiency was expected to be second to the meso Si NP samples due to the questionable quality of the Si/SiO_2_ interface and parasitic light absorption in SiO_2_ material both reducing the SHG efficiency. The nonannealed as-synthesized meso Si/SiO_2_ NP and meso SiO_2_ NP samples were expected to be the least efficient due to the absence of nc-Si phase and modest SHG efficiency of SiO_2_:OH^−^ material, which was reported in literature [63], [64], [65]. However, the SHG experiments revealed a picture that was divergent from these expectations.

To study nonlinear optical phenomena, 4 types of samples were considered: (i) meso Si NPs, (ii) SiO_2_ NPs, (iii) meso Si/SiO_2_ NPs with amorphous silicon in the pores, and (iv) annealed meso Si/SiO_2_ NPs with nc-Si in the pores. The pumping laser wavelengths were in the range of (910–960) nm, which corresponds to efficient light localization in the considered NPs (see Section S2 for details).

**Figure 3:**
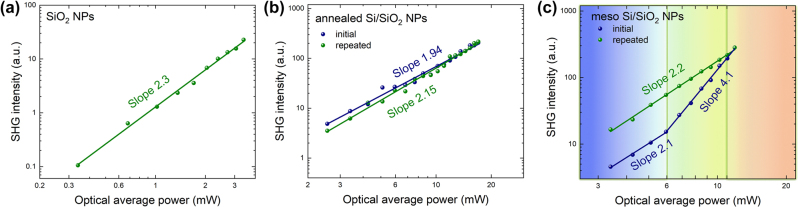
SHG-to-pump curves for (a) reference SiO_2_ NPs, (b) thermally annealed, and (c) as-synthesized meso Si/SiO_2_ NPs. For (b) and (c), the blue curves correspond to initial measurements, and green curves correspond to the repeated measurements. The colored areas in (c) present as follows: blue area – normal SHG response at relatively low pump power, green area – an increased slope on SHG response, pink area – irreversible sample damage at high pump power.

**Figure 4:**
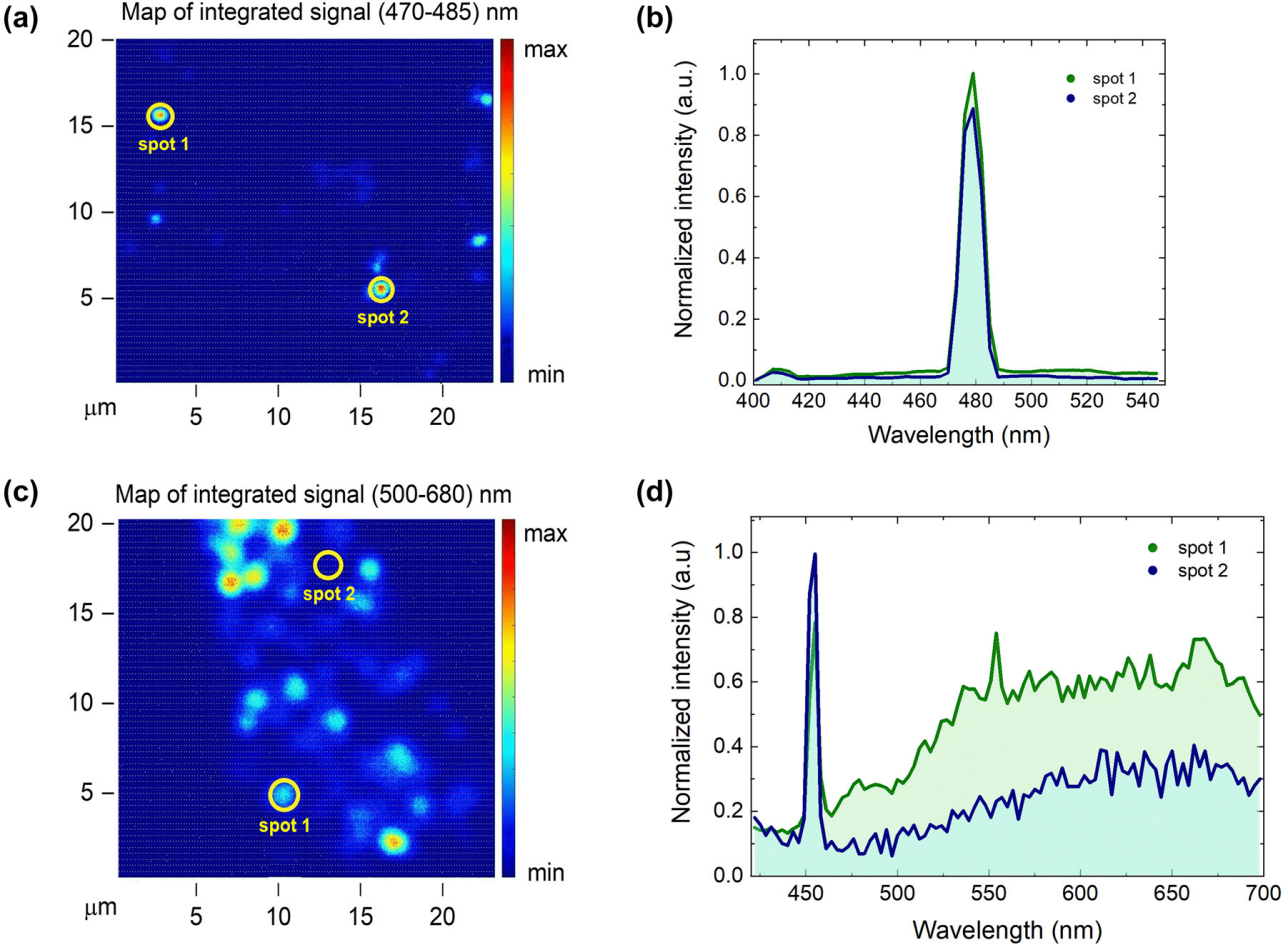
Optical response from (a, b) single reference SiO_2_ NPs under fs-laser excitation of 960 nm wavelength and (c, d) single meso Si/SiO_2_ NPs annealed under 910 nm wavelength fs laser with high power. (a) LSM map of reference SiO_2_ NP layers, integrated in (470–485) nm spectral range, corresponding to SHG signal, (c) LSM map of meso Si/SiO_2_ NP layers, integrated in (500–680) nm spectral range, corresponding to PL signal. (b, d) Corresponding spectral characteristics. Circles on LSM maps denote the positions of spectra acquisition.

The systematic study of meso Si NPs, both nonannealed and annealed, recently synthesized and aged, revealed a very weak SHG signal. We consider it might have been due to the structure damages during the chemical removal of SiO_2_ material from the blank meso Si/SiO_2_ NPs or the deteriorating effect of the inevitable formation of natural oxide layer under the SHG measurement conditions. In fact, pure meso Si NPs demonstrated negligible SHG signal in comparison with the reference SiO_2_:OH^−^ NPs. Apparently, the later demonstrated fairly distinguishable SH with an intensity-to-pump power ratio in a log-log scale (commonly referred in the literature as *slope*) close to the characteristic value of 2 (Figure 3a); the spectral characteristic at 960 nm IR fs excitation pulses is dominated by a narrow line at 480 nm (Figure 4b). Apart from the line attributed to the SH wavelength, the optical response for reference SiO_2_:OH^−^ NPs had no other distinguishable features in the studied region of 400–550 nm that indicates an absence of PL or other parasitic luminescence. A typical measured SHG map is presented in Figure 4a; the resolution allows distinguishing the individual SiO_2_:OH^−^ NPs. The inhomogeneous SHG intensity from different NPs is due to the different focusing since the NPs were not lying in the same plane.

The slope of 2.3 for reference SiO_2_:OH^−^ NPs (Figure 3a) is in full agreement with the data reported in the literature [65], [66], while the strong signal indicates a remarkably efficient SHG process that will be thoroughly studied elsewhere. It should be noted that the SiO_2_:OH^−^ NPs did not demonstrate significant changes of SH intensity due to the fs-laser annealing, i.e., the OH^−^ group-related SHG in SiO_2_ NPs is thermally stable.

The most efficient SHG was observed for the meso Si/SiO_2_ NPs. The thermally annealed meso Si/SiO_2_ NPs demonstrated a standard SH slope value of 2, and SH intensity-pump curves are reproducible at consequent measurements (Figure 3b). Interestingly, the as-synthesized (nonannealed) meso Si/SiO_2_ NPs demonstrated hysteresis in initial measurements of SH intensity-pump curves (Figure 3c), i.e., the firstly acquired curve was different from the consequent. We consider it is due to the *in situ* annealing of the meso Si/SiO_2_ NPs (with amorphous Si phase) under the probing IR laser pulses leading to the formation of nc-Si phase and possible changes of the Si/SiO_2_ interface. Indeed, the as-synthesized meso Si/SiO_2_ NPs demonstrated at the first measurement a significantly lower SHG signal compared to the thermally annealed meso NPs, while the slope was close to 2 before the threshold pump power value of about 3 mW (the corresponding optical power range is marked by a blue color in Figure 3c). After the threshold, the SH intensity started to improve rapidly that gave a superquadratic slope value of about 4.1, which can be associated with local annealing under fs laser irradiation (this power range is marked by green color in Figure 3c). It should be noted, that this effect is similar to the superquadratic hysteresis behavior previously reported for the laser pump-induced modifications of Si nanowire array [18]. SHG reached the maximum efficiency in the range of 10–12 mW pump power that is limited by the material degradation due to the overheating (the overheat power range is marked in Figure 3c by pink color). The measurements, repeated after the first acquisition, demonstrated a reproducible pump power dependency with a near-quadratic slope. Remarkably, the SH intensity value corresponding to the *in situ* annealed meso Si/SiO_2_ NPs is close to the performance of the meso Si/SiO_2_ NPs annealed in a sealed ampule.

Following the experimental observation of the SHG signal dependency on the pump power, the mesoporous Si/SiO_2_ nanoparticles undergo an ablation and the crystalline fraction of Si in the system starts depending on the input power. For moderately low intensities, the SH signal grows quadratically on the pump as the ablation process remains negligible. For higher intensities, however, the fraction of Si grows owing to ablation and, as a result, the effective polarizability of the nonlinear particle begins to depend on the input power, leading to 4th power dependence. Similar effects of power-dependent polarizability were observed in Ref. [67], where linear scattering spectra were investigated.

We proposed a model for ablation-dependent SHG, based on a single dipole. The dipole moment is given by: *p*_*L*_ = *α*_*dip*_ *E*_0_, while we suggest that the dipole polarizability (due to the recrystallization process) depends on the pumping intensity *α*_*dip*_ = *α*_0_ + *α*_1_ *I*_0_. Therefore, it gives subquadratic dependence at high excitation intensity (see Section S2 in the Supporting Information for details).

Since the energy of excitation photons (corresponding to 910–960 nm or 1.29–1.36 eV) is low compared to the energy bandgap of amorphous Si (around 1.7–1.8 eV [68], depending on methods of deposition), we suggest that the laser annealing can be caused by a multiphoton absorption process. Thus, at high fs laser intensity, multiphoton processes can give a significant contribution, which also explains the subquadratic behavior of SHG on laser intensity. At the same time, when the crystalline Si phase appears (energy band gap of 1.1 eV [69] and higher, depending on the size of crystallites), linear absorption can also take place.

Finally, we acquired spectral characteristics from individual meso Si/SiO_2_ NPs measured after annealing under the fs laser irradiation of 910 nm wavelength (Figure 4c and d). One can see that optical response demonstrates not only SHG signal (at 455 nm) but also a broad PL response in the range of (500–700) nm. This response becomes more prominent with an increasing laser irradiation power (Figure 4d), demonstrating the annealing of NPs and formation of nc-Si phase. The emergence of visible range PL indicates the presence of nanocrystalline silicon in Si/SiO_2_ structure within the pores of the mesoporous particle [70]. Moreover, the spectral positions of PL maxima indicate the average size of Si nanocrystals that is 3.1 nm in our case [53], [71], [72], [73]. This value is less compared to data acquired from Raman measurements for the thermally annealed meso Si/SiO_2_ NPs (Figure 2c), which indicates the difference in annealing regimes. Additional dark-field and linear PL measurements presented in Sections S3 and 4 in the Supporting Information also confirm the recrystallization phenomenon of silicon material in meso Si/SiO_2_ NPs under fs-laser illumination.

Theoretical calculations for light-induced heating in Si/SiO_2_ nanospheres, reported in Ref. [74], show that higher temperature can be achieved under laser irradiation in nanospheres consisting of amorphous Si (α-Si) compared to ones made of crystalline Si (c-Si). Due to the higher thermal conductivity of c-Si (compared to α-Si), better heat dissipation is observed in c-Si-based nanostructures in comparison with α-Si ones. Moreover, the enhanced optical absorption governed by optical resonances of nanostructures resulted in the increase of the local temperature compared to films or nonresonant nanostructures [75]. Thus, the local temperature in α-Si/SiO_2_ nanospheres under laser irradiation can reach values comparable with a melting point even at moderate laser intensity [74].

The reported theoretical results support our observation that the as-synthesized meso Si/SiO_2_ NPs, consisting of Si in the amorphous phase, can be efficiently heated by a laser. This heating promotes the Si phase transition from amorphous to crystalline. Moreover, this transition can occur locally within Si-based nanostructures, without its significant reshaping [75].

Thus, we consider that thermal annealing in the ampoule affects the crystallization of silicon in the pores of meso Si/SiO_2_ NPs almost in the same manner as the *in situ* laser-induced annealing, but the crystallization process using an LSM setup is controlled by knowing the reference value of the power, at which destruction of the material occurs. In the case of annealing under laser pulses, we control not only the power used to change the crystallization, but we can also understand how much silicon in the pore has undergone a change in its phase, in contrast to the annealing in an ampoule, when it is impossible to determine *a priori* the exact temperature and annealing time required for crystallization of the material in the pores.

## 4 Conclusions

In this work, SHG in mesoporous Si-based nanoparticles has been studied, where the enhancement of SHG in mesoporous Si/SiO_2_ nanoparticles has been demonstrated. The SHG efficiency has been found to be increased as the silicon in nanopores is annealed under femtosecond near-infrared pulses and transforms into the nanocrystalline phase. Remarkably, the SiO_2_ mesoporous frame of Si/SiO_2_ nanoparticles plays a dual positive role for the SHG process: first, it stabilizes the Si material; second, SiO_2_:OH^−^ material has a second-order nonlinearity itself and impacts to the observed SH signal. Moreover, Si/SiO_2_ nanoparticles emit light in a broad spectral range under fs-laser excitation, which can be employed for visualization purposes.

The proposed mesoporous Si/SiO_2_ nanoparticles can be considered as a very promising structure for SHG studies and applications at the nanoscale, since they combine most of the known approaches to facilitate SHG in silicon-based material (most notably: microcavity, nanocrystal phase, high specific surface area), while the fabrication method is scalable and cost-effective.

## Supplementary Material

Supplementary Material Details
