# Supplementary material for: Second harmonic generation and broad-band photoluminescence in mesoporous Si/SiO2 nanoparticles
Source: Nanophotonics. 2024 Aug 1;13(18):3299–309. doi: 10.1515/nanoph-2024-0218 (PMC11501142; doi:10.1515/nanoph-2024-0218)
Supplement: Supplementary file 1 — Supplementary Material Details [file j_nanoph-2024-0218_suppl_001.pdf]

Supporting Information

Second harmonic generation and broad-band photoluminescence in mesoporous Si/SiO_2 nanoparticles

Viktoriia Mastalieva[1,2,*], Vladimir Neplokh[1,3], Arseniy Aybush[4], Ekaterina Stovpiaga[2], Daniil Eurov[2], Maksim Vinnichenko[3], Danila Karaulov[2,3], Demid Kirillenko[2], Alexey Mozharov[1], Vladislav Sharov[1,2], Denis Kolchanov[5], Andrey Machnev[5], Valery Golubev[2], Alexander Smirnov[2], Pavel Ginzburg[5], Sergey Makarov[6,7], Dmitry Kurdyukov[2], Ivan Mukhin[1,3,6]

[1] Alferov University, Khlopina 8/3, 194021, St. Petersburg, Russia
[2] Ioffe Institute, 194021, Russia, St. Petersburg, Polytechnicheskaya str., 26
[3] Peter the Great St. Petersburg Polytechnic University, Polytechnicheskaya 29, 195251, St. Petersburg, Russia
[4] N.N. Semenov Federal Research Center for Chemical Physics, Russian Academy of Sciences, Kosygin Street 4, 119991 Moscow, Russia
[5] Tel Aviv University, Ramat Aviv, 69978 Tel Aviv, Israel
[6] Qingdao Innovation and Development Center, Harbin Engineering University, Qingdao 266000, Shandong, China
[7] ITMO University, 197101, St. Petersburg, Russia

*corresponding author email: *strindberg76@mail.ru*

S1. Scanning electron microscopy of Si/SiO_2 nanoparticles

Representative scanning electron microscopy (SEM) images of the Si/SiO_2 nanoparticles (NPs) before and after thermal annealing are presented in Figure S1. One can see that the NPs survive and preserve their shape after thermal treatment.

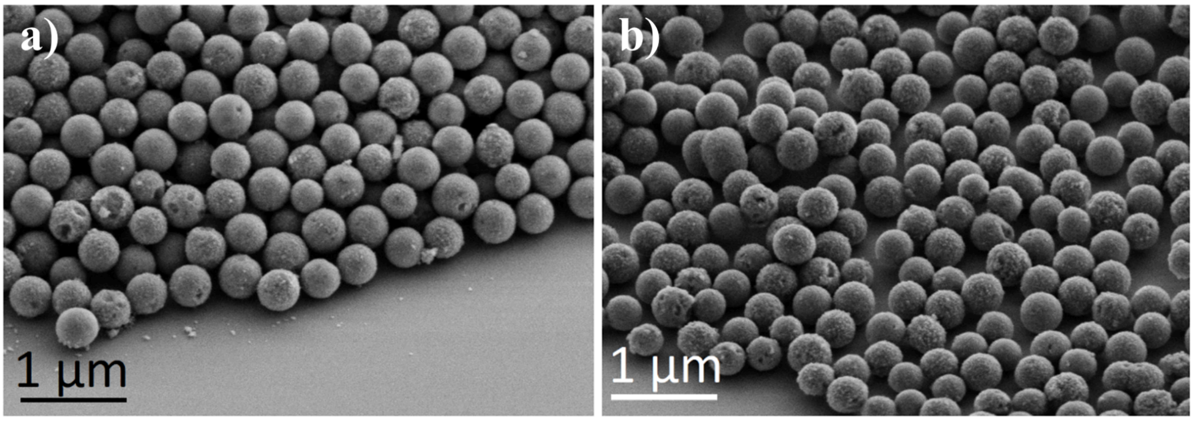

Figure S1. SEM images of composite Si/SiO_2 particles before (a) and after (b) thermal annealing.

S2. Theoretical description of optical response

First, we describe an ablation-dependent second harmonic generation (SHG) of Si/SiO_2 nanoparticles. Following the experimental observation of the second harmonic signal dependency on the pump power, the following theoretical model has been proposed. The mesoporous Si/SiO_2 nanoparticle undergoes an ablation and the crystalline fraction of Si in the system starts depending on the input power. For moderately low intensities, the second harmonic signal grows quadratically on the pump as the ablation process remains negligible. For higher intensities, however, the fraction of Si grows owing to ablation and, as a result, the effective polarizability of the nonlinear particle begins to depend on the input power, leading to 4th power dependence. Similar effects of power-dependent polarizability were observed in [1], where linear scattering spectra were investigated. Hereinafter, we propose a simple model for ablation-dependent SHG, based on a single dipole.

The dipole moment of a dipole is given by:

$$p_L = \alpha_{dip} E_0 \quad (1)$$

where p_L is the linear dipole moment, $\alpha_{dip} = 3\varepsilon_0 V \frac{\varepsilon_m - 1}{\varepsilon_m + 2}$ is the dipole polarizability, ε_m and ε_0 is a material and vacuum permittivity, respectively, V is the volume of the spherical particle, and E_0 is the incident electric field.

The ablation is introduced via a dependence of α_{dip} on the incident intensity (I_0):

$$\alpha_{dip} = \alpha_0 + \alpha_1 I_0 \quad (2)$$

Here, α_0 is the linear polarizability component, and α_1 represents the coefficient of the power-dependent term. The nonlinear dipole moment p_{NL} is proportional to the square of the linear. This model, as a mere approximation, can encapsulate both the volume and surface nonlinearities of a real nanoparticle.

$$p_{NL} \sim \left|p_L\right|^2 = \left|\alpha_{dip}\right|^2 \left|E_0\right|^2 \quad (3)$$

The intensity of the second harmonic generation I_{SH} is then approximated as the square of the nonlinear dipole moment:

$$I_{SH} \sim \left|p_{NL}\right|^2 = \left|\alpha_{dip}\right|^4 \left|I_0\right|^2 \quad (4)$$

This relationship governs the SHG law.

The dependence of the second harmonic intensity on the pump intensity appears in Figure S2. The parameters in Eq. 4 were fitted to correspond to the breakpoint (between quadratic and cubic behavior), found in the experiment - Figure 3(c) in the main manuscript. The breakpoint is observed at ~6 mW and, taking the parameters of the laser of an optical system, it corresponds to $3 \times 10^{7} W/m^{2}$ peak intensity. For calculating the linear polarizability, a SiO_2 particle with a radius of 450 nm was considered. From Figure S1, the value of the α_1 of $\sim 3.532\, m^{5}/W$ can be deduced.

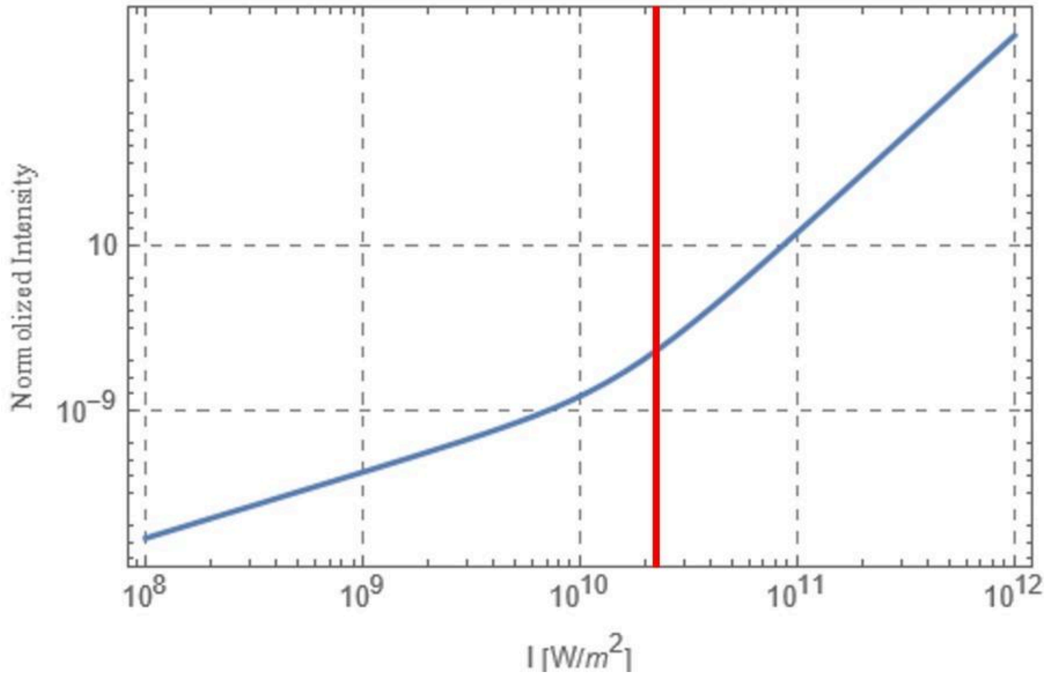

Figure S2. SHG intensity as the function of the pump intensity (logarithmic scales). The breakpoint, indicating the change in slopes between 2 and 4 is marked with a red line (at $3 \times 10^{7} W/m^{2}$).

To reveal the field structure inside the particle, we calculated the electrical field distribution within the pure SiO_2 and mesoporous Si/SiO_2 NP (see the considered geometry in the insert in Figure S3), applying finite element modeling techniques.

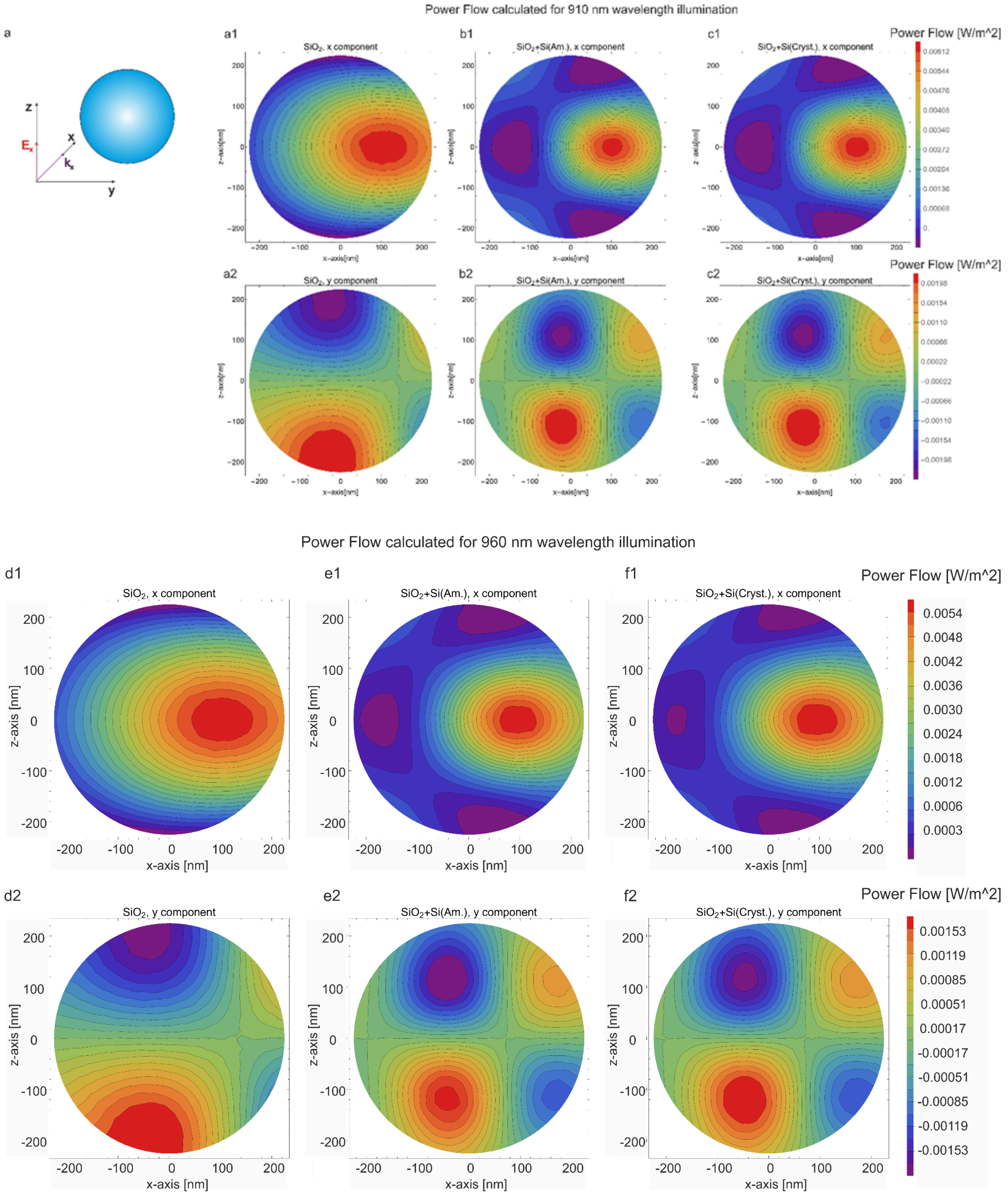

Figure S3. (a) Schematic representation illustrating the considered geometry. The incident plane wave propagates towards the NPs along the x-axis, with polarization parallel to the z-axis. The calculated power flow, including both x and y components, across three distinct NPs: SiO_2 (a1, a2, d1, d2), Si/SiO_2 in the amorphous Si phase (b1, b2, e1, e2), and Si/SiO_2 in the crystalline Si phase (c1, c2, f1, f2).

Figure S3 presents the calculated power flow over the particle volume, within pure SiO_2 particle and Si/SiO_2 particle with 50% filling by volume, modeled for two wavelengths of 910 nm and 960 nm. The wavevector k_o of the propagating wave is parallel to the X-axis. The calculated 2D plots of both components of the power flow correspond to the cross-sections in (XZ) plane cutting through the center of the sphere. Figure S3(a1, a2, d1, d2) show results for pure silica particles, while Figure S3(b1, b2, e1, e2) correspond to silica particles containing amorphous silicon (a-Si) inclusions, and Figure S3(c1, c2, f1, f2) depict silica particles with crystalline silicon (c-Si) inclusions. The effective refractive index was estimated using the Maxwell-Garnett formula, relying on the effective medium approximation [2]. The values lie between $n_{SiO2} < n_{eff} < n_{Si}$. However, it strongly depends on the filing factor parameter f, reflecting the NP porosity. In our case, we considered the feeling factor of 5% for SiO_2 particles and 50% for meso Si/SiO_2 particles. For example, for SiO_2 particles filled with amorphous silicon nanoinclusions, the effective refractive index is about 1.5277 at a 910 nm wavelength. One can see that filling the pores with Si increases the effective refractive index (n) of NPs and as a result higher electric field localization for both wavelengths. Due to close values of n for a-Si and c-Si [3, 4], the field distribution does not significantly depend on the crystalline phase of silicon inclusions.

As reported in [5], AlGaAs cylinders having dimensions around 400-450 nm support the magnetic and electric optical modes (both dipole and quadrupole) for wavelength exceeding 1 μm. At the same time, pure Si NPs with a 450 nm diameter have electric quadrupole mode near 936 nm. However, the refractive index of the considered AlGaAs composition and Si are equal to around 3.5-3.6, which is significantly higher compared to our mesoporous nanoparticles (1.4, 2.3, and 2.5 for meso SiO_2, Si and Si/SiO_2 NPs, respectively). Therefore, we didn't expect the significant contribution of higher multipoles, and the main scattering contribution is governed by an electric dipole for the studied spectral range (910-960 nm for excitation wavelengths and 455-480 for SHG response) - see the results of dark-field scattering spectra measurements for single mesoporous Si/SiO_2 NP (Figure S4 b).

Moreover, the studied meso Si and Si/SiO_2 NPs were approximately in the same resonance conditions at the excitation wavelengths, which allowed us a direct comparison of the nonlinear response of the studied nanomaterials. We would like to point out that in our work we didn't study the optical response from pure Si NPs, having higher refractive index (~3.6), and focused on mesoporous Si NPs half-filled by air or silica.

S3. Dark-field measurements of meso Si/SiO_2 NPs

To experimentally prove a crystalline phase transition in the amorphous part of mesoporous Si/SiO_2 NPs we performed optical dark field measurements.

Dark field microscopy was conducted using a Leica DMi8 inverted microscope, equipped with a high numerical aperture (NA) dark field condenser of 0.95 NA. We captured light scattering from the sample with a 100x magnification objective lens (0.85 NA). The scattered

light was then directed through the microscope's eyepiece into an Avantes fiber-optic spectrometer for precise spectral analysis.

Femtosecond laser ablation, performed using a MENLO YLMA laser with a pulse duration of 100 fs and variable optical power up to 60 mW, was applied to the specimen for amorphous silicon recrystallization. An observed decrease in the scattering spectra with increased laser output power was consistent with the recrystallization of silicon, transitioning from an amorphous to a crystalline phase.

A typical SEM image of single Si/SiO_2 NPs on an axillary substrate with gold marks is demonstrated in Figure S4 a). Figure S4 b) shows dark-field scattering spectra obtained from a single mesoporous Si/SiO_2 NP, subjected to fs laser heating with different laser power. One can see that increasing the ablation power leads to a decrease in scattering amplitude. This can be associated with a decrease in the effective refractive index of NP. Indeed, the refractive index of amorphous Si is higher than crystalline one [3, 4]. Considering the preservation of the NP size under temperature treatment, the observed results reveal the recrystallization of the Si part of the studied mesoporous Si/SiO_2 NPs under fs laser ablation. A similar phenomenon was observed for laser-induced crystallization of amorphous Si nanoparticles [6].

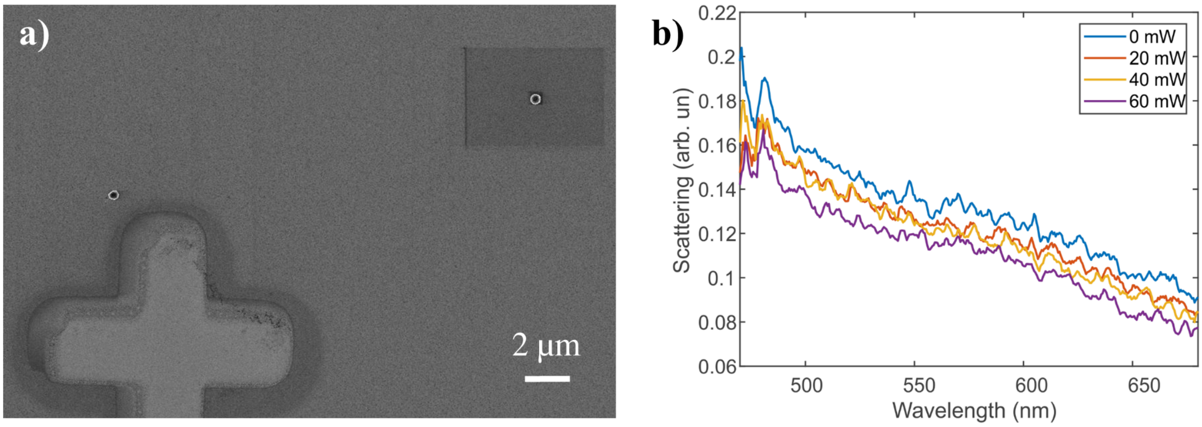

Figure S4. a) SEM image of single Si/SiO_2 NPs located near the gold alignment mark. b) Dark-field scattering spectra of single mesoporous Si/SiO_2 NP additionally irradiated with fs-laser with different power levels (in legends).

S4. Linear photoluminescence measurements of meso Si/SiO_2 NPs

We performed linear photoluminescence (PL) measurements for both thermally annealed and as-synthesized (non-annealed) Si/SiO_2 NP samples with a 532 nm laser excitation that confirmed the presence of nc-Si PL (Figure S5).

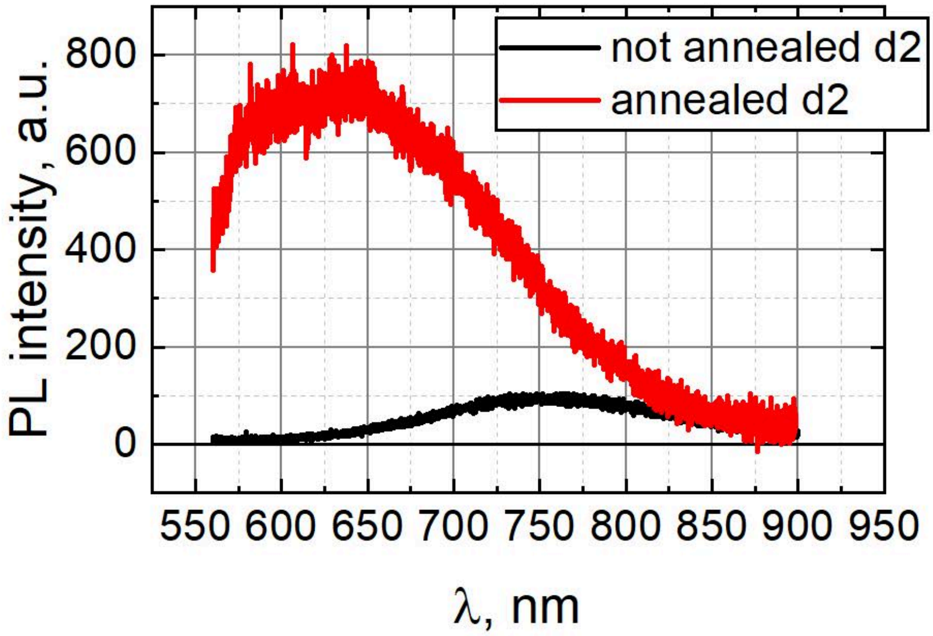

Figure S5. PL spectra at linear 532 laser excitation of composite Si/SiO_2 nanoparticles before (black line) and after (red line) thermal annealing.

One can see that the acquired linear PL response corresponds well to the data shown in Figure 4 d) of the main manuscript. The slight difference can be associated with different annealing regimes for the investigated NPs (thermal and fs laser-induced annealing). The obtained data confirms the presence of nc-Si phase in the considered Si/SiO_2 NPs.

References:

[1] U. Zywietz, A. B. Evlyukhin, C. Reinhardt, and B. N. Chichkov, "Laser printing of silicon nanoparticles with resonant optical electric and magnetic responses," *Nat. Commun. 2014 51*, vol. 5, no. 1, pp. 1–7, Mar. 2014, doi: 10.1038/ncomms4402.

[2] Koledintseva, M., DuBroff, R., & Schwartz, R. (2006). A Maxwell Garnett model for dielectric mixtures containing conducting particles at optical frequencies. Progress in electromagnetics research, 63, 223-242.

[3] Green, M. A. (2008). Self-consistent optical parameters of intrinsic silicon at 300 K including temperature coefficients. Solar Energy Materials and Solar Cells, 92(11), 1305-1310.

[4] Pierce, D. T., & Spicer, W. E. (1972). Electronic structure of amorphous Si from photoemission and optical studies. Physical Review B, 5(8), 3017.

[5] Carletti, L., Locatelli, A., Stepanenko, O., Leo, G., & De Angelis, C. (2015). Enhanced second-harmonic generation from magnetic resonance in AlGaAs nanoantennas. *Optics express*, *23*(20), 26544-26550.

[6] Zywietz, U., Evlyukhin, A. B., Reinhardt, C., & Chichkov, B. N. (2014). Laser printing of silicon nanoparticles with resonant optical electric and magnetic responses. Nature communications, 5(1), 3402.
